# The role of meal viscosity and oat β-glucan characteristics in human appetite control: a randomized crossover trial

**DOI:** 10.1186/1475-2891-13-49

**Published:** 2014-05-28

**Authors:** Candida J Rebello, Yi-Fang Chu, William D Johnson, Corby K Martin, Hongmei Han, Nicolas Bordenave, Yuhui Shi, Marianne O’Shea, Frank L Greenway

**Affiliations:** 1 Pennington Biomedical Research Center, Louisiana State University System, 6400 Perkins Road, Baton Rouge, LA 70808, USA; 2 PepsiCo R&D Nutrition, 617 W. Main Street, Barrington, IL 60010, USA

**Keywords:** Appetite, β-glucan, Oats, Viscosity, Physicochemical properties

## Abstract

**Background:**

Foods that enhance satiety can help consumers to resist environmental cues to eat, and improve the nutritional quality of their diets. Viscosity generated by oat β-glucan, influences gastrointestinal mechanisms that mediate satiety. Differences in the source, processing treatments, and interactions with other constituents in the food matrix affect the amount, solubility, molecular weight, and structure of the β-glucan in products, which in turn influences the viscosity. This study examined the effect of two types of oatmeal and an oat-based ready-to-eat breakfast cereal (RTEC) on appetite, and assessed differences in meal viscosity and β-glucan characteristics among the cereals.

**Methods:**

Forty-eight individuals were enrolled in a randomized crossover trial. Subjects consumed isocaloric breakfast meals containing instant oatmeal (IO), old-fashioned oatmeal (SO) or RTEC in random order at least a week apart. Each breakfast meal contained 218 kcal (150 kcal cereal, and 68 kcal milk) Visual analogue scales measuring appetite were completed before breakfast, and over four hours, following the meal. Starch digestion kinetics, meal viscosities, and β-glucan characteristics for each meal were determined. Appetite responses were analyzed by area under the curve. Mixed models were used to analyze response changes over time.

**Results:**

IO increased fullness (p = 0.04), suppressed desire to eat (p = 0.01) and reduced prospective intake (p < 0.01) more than the RTEC over four hours, and consistently at the 60 minute time-point. SO reduced prospective intake (p = 0.04) more than the RTEC. Hunger scores were not significantly different except that IO reduced hunger more than the RTEC at the 60 minute time-point. IO and SO had higher β-glucan content, molecular weight, gastric viscosity, and larger hydration spheres than the RTEC, and IO had greater viscosity after oral and initial gastric digestion (initial viscosity) than the RTEC.

**Conclusion:**

IO and SO improved appetite control over four hours compared to RTEC. Initial viscosity of oatmeal may be especially important for reducing appetite.

## Introduction

The prevalence of obesity has increased in every region of the world, including several countries with low and middle incomes [1]. Evidence for a leveling off in the steep rises previously observed in high income countries, however, does not amount to a reversal of the obesity epidemic [2]. The US will have a projected 65 million more obese adults in 2030 compared to 2010 [3]. Reversing this epidemic requires developing effective ways of curbing excessive energy intake. The role of dietary fiber in promoting satiation and satiety has been the focus of a vast amount of research, and there is evidence to indicate that consumption of fiber-rich foods has a modest long-term effect on weight loss [4,5].

Dietary fiber is classified as soluble and insoluble fiber based on its solubility in aqueous enzyme solutions similar to those in the gastrointestinal tract [6]. Some soluble fibers such as β-glucan form a viscous solution when mixed with liquids. Viscosity is an important rheological property of β-glucan, and is associated with beneficial physiologic responses that mediate appetite regulation such as delayed gastric emptying, increased stomach distension, and delayed intestinal transit [7]. The increased viscosity of intestinal contents prolongs transit time and the absorption rate of nutrients [8]. A thickening of the unstirred water layer in the intestine further impedes absorption [9]. Enhanced interaction between nutrients and the intestinal mucosa stimulates the release of appetite regulating peptides which either function distantly as hormones or activate nearby nerve fibers [10]. Some afferent neurons in the gastric mucosa are mechanoreceptors while others may transduce chemical or other signals of satiation [11]. Gastric and intestinal signals may also work in synergy to influence appetite [11].

When making choices of foods rich in fiber to enhance satiety it is also important to consider the manner in which the food is processed. β-glucan, is abundant in oat kernels and exhibits a high viscosity at relatively low concentrations [12]. However, the processing of oats, the concentration of soluble fiber, and the processing of products containing β-glucan affect the amount, solubility, molecular weight, structure, and functionality of β-glucan [12,13]. Kilning, a hydrothermal treatment used in the processing of oats induces structural changes in the oats, as do the high shear forces of the flaking process. Old fashioned oats (SO) and instant oats (IO) have different kilning and flaking processes. Further, unlike old-fashioned oats instant oats are cut which exposes the endosperm and affects the β-glucan, a major component of endosperm cell walls. In studies that investigated the effects of β-glucan on satiety, data on the physicochemical properties of the fiber are singularly lacking [14-18]. Thus, investigating the physicochemical properties of the fiber would help clarify the mechanisms by which β-glucan affects satiety, the amount of β-glucan that elicits an appetite response, and the processing techniques that could facilitate development of satiety-enhancing products to replace foods in the usual diet.

In a previous study [19] we demonstrated that a breakfast meal containing 250 kcal serving of SO increased satiety compared to an isocaloric meal containing 250 kcal of the most widely consumed oat-based ready-to-eat breakfast cereal (RTEC) in the United States ((based on IRI Liquid Data, 52 Weeks Ending March 11, 2012). The present study measured the satiety effect of breakfast meals containing single servings (150 kcal) of oatmeal (IO and SO) and an isocaloric serving of the RTEC over four hours. Instant oats and SO differ in the manner in which they are processed; hence, in addition to portion size, the purpose of the present study was to determine if differences in processing influence the outcome. Since replacing foods in the usual diet with foods that increase satiety may be a means of reducing energy intake, a popular oat-based RTEC was used as a comparator even though it differed in nutrient composition and β-glucan content. The meal viscosity, starch digestion kinetics, and β-glucan characteristics of each breakfast meal were also determined. It was hypothesized that both the IO and SO breakfast meals would have a higher viscosity and would increase satiety more than the RTEC.

## Methods

### Subjects

Forty-eight healthy subjects 18 years of age or older were enrolled in a randomized, three treatment, crossover trial. All subjects participated in an initial screening that involved measurement of body weight; height; waist and hip circumferences; vital signs (blood pressure and pulse rate); chemistry-15 panel (glucose, creatinine, potassium, uric acid, albumin, calcium, magnesium, creatine phosphokinase, alanine aminotransferase, alkaline phosphatase, iron, cholesterol [total, high density lipoprotein, low density lipoprotein], and triglycerides); complete blood count with differential; and beta-human chorionic gonadotropin urine pregnancy test (in females of child-bearing potential). Health was further assessed through the administration of a medical screening questionnaire. Female subjects also completed a menstrual cycle questionnaire to ensure that test days would fall within the luteal phase of the menstrual cycle [20]. Inclusion criteria were: (i) healthy individuals taking no medication other than birth control or hormone replacement (ii) Willing to use an effective method of birth control during the course of the study, if female and capable of bearing children. Exclusion criteria were: (i) women who were pregnant or nursing, (ii) self-reported weight gain or loss of ≥ 4 kg in the last 3 months, (iii) fasting glucose >126 mg/dL, (iv) dietary restraint score ≥ 14, as assessed by the Dietary Restraint Scale of the Eating Inventory [21] and (v) allergy or intolerance to oats or milk.

This study was approved by the Institutional Review Board of the Pennington Biomedical Research Center, Baton Rouge, where the study was conducted. Participants provided written informed consent. The trial was registered on ClinicaTrials.gov with registration number NCT01666561. Recruitment for clinical trials conducted at The Pennington Biomedical Research Center is coordinated by the Recruitment Core which is responsible for the design and placement of advertisements, and screening of all incoming requests to determine study eligibility. Participants were recruited from Baton Rouge and the surrounding areas.

### Study design

The test breakfast meals consisting of Quaker Instant Oatmeal Flakes™ (IO), Quaker Old Fashioned Oatmeal™ (SO), and Honey Nut Cheerios™ (RTEC), were served to participants on three test days, separated by at least a week. There were six possibilities for assigning the order of the cereals for the three visits (abc, acb, bac, bca, cab, cba). Each subject was randomly assigned to one of these orders so that eight subjects were assigned to each order. The randomization was done by the study statistician and participants were enrolled by the study coordinator. The study dietitian who had no interaction with study subjects provided the test meals for the participants and had sole access to the random assignment until data analysis. The breakfast meals contained 217.5 total kcal, consisting of 150 kcal from the cereal, and 67.5 kcal from lactose-free, fat-free milk. The old fashioned oatmeal, (one standard serving, 40 g dry weight), was prepared by adding one cup room temperature water (240 g) and microwaving at high power for three minutes. The instant oatmeal (40 g dry weight) was stirred, following the addition of one cup boiling water. Both were allowed to stand for one minute, and were served with 184.2 g of cold milk. The RTEC (38.2 g dry weight), was prepared by adding 184.2 g of cold milk, and was served with 240 g of water. The participants had the option of adding one g of Splenda™ and one-half teaspoon of cinnamon to the oatmeal. If the participant added the Splenda™ and cinnamon to the oatmeal, they were required to add the same amounts of both to the RTEC.

At each test breakfast visit, participants arrived at the center after fasting (except for water) for 10 hours overnight. They were also required to avoid alcohol and strenuous exercise for 24 hours prior to the test meal. To determine the presence of colds or allergies that might affect taste, participants were required to complete a questionnaire and were asked to return on another day if such a condition was present. Prior to serving the test meal hunger, fullness, desire to eat, and prospective intake were assessed using electronic visual analog scales (VAS) [22,23]. Participants rated each subjective state on a continuous line that was anchored using the descriptors ‘Not at all’ to ‘Extremely’, and displayed on a computer screen. Visual analog scales were scored by the computer on a 0 to 100 unit scale and the score was sent directly to the database. Hunger, fullness, desire to eat, and prospective intake, were assessed. The subjects were presented with their first breakfast test and given 20 minutes to eat it. Test meals were supervised to ensure that the entire breakfast was eaten. Visual analog scales were then administered at 30, 60, 120, 180, and 240 minutes following the start of the breakfast meal and subjects were asked an open ended question (How do you feel?) at each of these time-points to elicit any adverse events. Subjects were required to remain in the dining area during this period, and refrain from any food or drink.

### Kinetics of in vitro glucose release

Oatmeal was prepared as described in the study design, and allowed to rest for one minute. This oatmeal and dry RTEC were first analyzed for their sugar content by high performance liquid chromatography and total starch content by standard American Association of Cereal Chemists (AACC) procedures [24]. Thus, their total glucose content (G_tot_) was determined. The cereals then underwent a three-stage *in vitro* digestion procedure described by Sopade and Gidley [25] to evaluate the kinetics of glucose release. The aim of this method which has been used extensively in the literature [26-28] is to explore differences in digestibility of starches caused by differences in their physicochemical and structural characteristics. During this procedure, α-amylase, pepsin, and pancreatin with amyloglucosidase were added in a timely order with adjustments to their corresponding working pH to mimic digestion in the oral cavity, stomach, and small intestine. Glucose release from about 500 mg of digested food in the simulated small intestine phase was monitored over the course of three hours using the Accu-Check Aviva glucometer (Roche Diagnostics, Indianapolis, IN) which was previously calibrated for glucose response and interactions with other sugars potentially present in the matrix. Data were compiled as digestograms (G_t=time_ × 100/G_tot_ versus time). Digestograms were fitted using a first order kinetic law, from where initial glucose release (glucose concentration at the beginning of the intestinal phase [G_t=0_]), rate of glucose release (time constant of the first order law) time of half-of-total glucose release (time for G_t_ × 100/G_tot_ = 50%), and area under the curve (AUC) were calculated. Area under the curve values were normalized with a white bread control (AUC_white bread_ = 100).

### Characterization of β-glucan

The RTEC, SO, and IO, were ground to flour. The β-glucan component was extracted from dry ground oat flakes and RTEC, according to the procedure described by Rimsten *et al*. [29]. After extraction, dialysis purification and drying, the β-glucan was subjected to molecular weight, distribution, and radius of gyration analysis using high performance size exclusion chromatography with multi angle light scattering detection (sample preparation: dissolution at 0.1% w/w in mobile phase; mobile phase: 50 mM NaNO_3_ and 200 ppm NaN_3_ in deionized water; flow rate: 0.8 mL.min^−1^; columns: Agilent PL aquagel-OH MIXED-H 8 μm 300 × 7.5 mm column and 50 × 7.5 mm guard column; Light Scattering detector: Wyatt Dawn Heleos II; Differential Refractive Index detector: Wyatt Optilab T-rEX, Wyatt Technology Europe GmbH, Dernbach, Germany). Molecular weight and radius of gyration calculations (parameters: second order Berry Model; dn/dc value: 0.146 mL.g^−1^) were performed using Wyatt Astra V software (Wyatt Technology, Santa Barbara, California).

The β-glucan content was measured using standardized AACC procedures [30]. Briefly, β-glucan was hydrolyzed by lichenase into oligosaccharides, which were converted into glucose by β-glucosidase. The amount of glucose released was measured by UV absorbance with glucose oxidase/peroxidase. The β-glucan content was calculated based on the amount of released glucose.

### Breakfast viscosity

The viscosity of the two breakfast meals was measured in a process simulating the gastric phase of digestion *in vivo*. A 250 kcal serving of each breakfast meal (66.8 g dry weight of oatmeal and 63.6 g dry weight of RTEC) was prepared as described in the study design, using proportionate amounts of liquids. Although the *in vitro* experiment tested a larger serving than the portion size used in the study, the relative effects are expected to be the similar, since the same ratio of cereal to liquid was used. Each sample underwent the first two phases (oral and gastric) of the three-stage *in vitro* digestion procedure described by Sopade and Gidley [25] and modified as follows: 61.2 mL of artificial saliva (250 U.mL^−1^ α-amylase in carbonate buffer at pH 6) was used for the oral phase, and 334 mL of a 1 mg.mL^−1^ pepsin in 0.02 M HCl was used for the gastric phase, for each sample. The viscosity of oatmeal and the RTEC was measured in triplicate at 0, 30, 60, 90, and 120 minutes in a 1000 mL beaker by using a viscometer (viscometer: Brookfield DV-I+; vane spindle #71; speed: 100 rpm [Brookfield, Middleboro, Massachusetts]).

### Statistical analysis

A mixed model analysis of variance for a crossover trial was performed to analyze the primary outcome. A strength of the crossover design is that significance of differences among treatments is evaluated in terms of pooled within subject comparisons. In parallel arm trials, pre-randomization covariates such as gender, age, and body mass index (BMI), can be included in analytical models to explain extraneous variability in outcome variables and increase precision in estimators of treatment effects. In cross-over trials pre-randomization covariates can influence only comparisons between groups containing different subjects in contrast to within subject comparisons where they have no influence on estimators of treatment effects or their precision [31]. Hence, covariates were not included in the models employed in this analysis.

Visual analog scale scores for hunger, fullness, desire to eat, and prospective food intake, were assessed at baseline and at 30, 60, 120, 180, and 240 minutes following the start of the breakfast meal. The AUC for each assessment was estimated using the linear trapezoidal rule and calculated as the area between the zero change line and the measured change curve which could be either above or below the zero change line. The statistical model included fixed effects (treatment sequence effects [residual treatment carryover effects from test day 1 to test day 2 and test day 2 to test day 3], test day main effects, and treatment main effects) and random effects (subjects within treatment sequence groups). Scores at each assessment time were analyzed using a mixed model ANOVA for a doubly repeated-measures crossover trial, where the first repeated measures variable was the test day, and the second was the time since start of breakfast. Any baseline differences in VAS scores among treatments were normalized to zero, and the resulting changes from baseline were summarized as least squares means plotted for each cereal type across the assessment times. The p-values were adjusted for multiple comparisons using the Bonferroni method. Differential treatment effects with respect to AUC and per time point were compared using SAS (version 9.2, 2002–2008, PROC MIXED; SAS Institute, Cary, NC).

Significance of among-treatment differences in viscosity, molecular weight, and radius of gyration was assessed using ANOVA. Values were expressed as means ± standard error. The AUC was used to analyze the kinetics of starch digestion and glucose release and the values were expressed as means ± standard deviation. Statistical significance was set at p < 0.05.

During the planning phase of the study, sample size was estimated using G*Power, Version 3.1.2 (F. Faul, Universitat Kiel, Kiel, Germany) with the following assumptions: (i) power ≥ 0.75 was considered acceptable, (ii) the significance level under the null hypothesis was set at α = 0.05, (iii) the primary outcome was VAS AUC with *a priori* standard deviation assumed to be 3047 mm × min based on previous research [32] and (iv) the null hypothesis was to be tested against a two-directional alternative. The study was sufficiently powered with 43 participants for detecting a minimum difference of 1258 mm × min between cereal types, which is similar to observed differences in AUC (1213 mm × min) for desire to eat from a similar study that assessed appetite sensations [32].

## Results

Forty eight subjects were enrolled in the study, of which three were underweight (BMI < 18.5), 22 were normal weight (BMI from 18.5 to 24.9), nine were overweight (BMI from 25 to 29.9) and 14 were obese (BMI > 30). Five participants were unable to complete the study. Two participants moved out of the area, one participant had difficulty in obtaining transportation to the Center, one participant had a conflicting schedule, and one participant had a change of mind. There were no adverse events. Descriptive characteristics of the subjects at baseline are summarized in Table 1. A nutrient analysis of the breakfast meals obtained from the nutrition facts label, and the β-glucan content which was measured are presented in Table 2.

**Table 1:** Subject characteristics at baseline including age, height, weight, body mass index, waist circumference, and gender

	**n = 48**	
	**Mean**	**Standard deviation**
Age	29.8	9.9
Height (cm)	167.2	9.9
Weight (kg)	75.7	19.5
Body Mass Index (kg/m^2^)	27.1	6.7
Waist Circumference (cm)	85.4	15.1
	n (%)	
Gender		
Female	28 (58.3)	
Male	20 (41.7)	

**Table 2:** Energy and nutrient content of breakfast meals

	SO^*^	**IO**^**†**^	**RTEC‡**	**Lactose-Free, Fat-Free Milk**
Energy (kcal)	150	150	150	67.5
Fat (g)	3.0	3.0	2.1	0
Protein (g)	5.0	5.0	2.7	6.0
Total Carbohydrates (g)	27.0	27.0	30.0	9.8
Total Fiber (g)	4.0	4.0	2.7	0
Soluble Fiber (g)	2.0	2.0	1.1	0
β-Glucan (g)	1.6	1.6	1.0	0
Sugar (g)	1.0	1.0	12.3	9.0
Sodium (mg)	0	0	218.3	93.8
Serving Size (g)	40	40	38.2	184.2

^*^Quaker Old Fashioned Oatmeal; (Pepsico Inc.Barrington IL).

^†^Quaker Instant Oatmeal Flakes; (Pepsico Inc.Barrington IL).

^‡^Honey Nut Cheerios; (General Mills Inc. Minneapolis MN).

### Hunger and fullness

**Figure 1:**
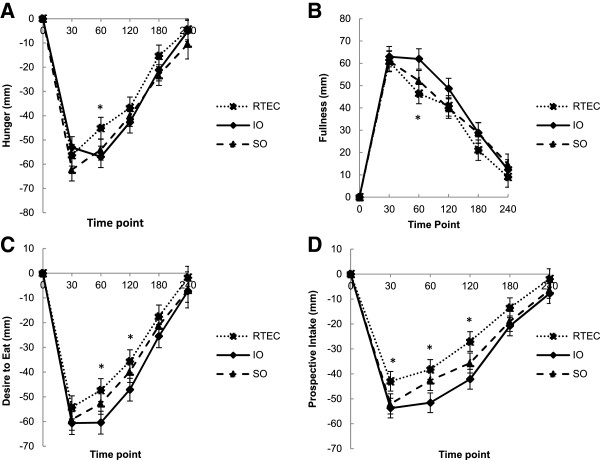
**Visual analog scale ratings for hunger (n = 48) before and after consumption of instant oatmeal (IO), old fashioned oatmeal (SO) and a ready-to-eat breakfast cereal (RTEC). (A)** Differences in hunger ratings among the three breakfast cereals as assessed by AUC were not statistically significant. *Least squares mean was different between IO and the RTEC at 60 minutes (p = 0.04). **(B)** Fullness ratings were different between IO and the RTEC by AUC. *Least squares mean was different between IO and the RTEC at 60 minutes (p < 0.01). **(C)** Desire to eat ratings were different between IO and the RTEC by AUC. *Least squares means were different between IO and the RTEC at 60 minutes (p < 0.01) and 120 minutes (p < 0.02). **(D)** Prospective intake ratings were different between the two types of oatmeal and the RTEC by AUC. *Least squares means were different between IO and the RTEC at 30 minutes (p < 0.02), 60 minutes (p < 0.01), and 120 minutes (p < 0.01).

The four hour AUC for VAS ratings of hunger were not statistically different among the three breakfast cereals; however, IO consumption reduced hunger at 60 minutes significantly more than the RTEC (p = 0.01) (Figure 1A). IO consumption increased fullness significantly more than the RTEC over the four-hour period following the meal (AUC IO: 9660.62 ± 885.5 mm × min versus RTEC: 7897.65 ± 887.65 mm × min, p = 0.04) and at 60 minutes (p < 0.01). IO consumption increased fullness more than SO at 60 minutes (p = 0.04), although fullness was not significantly different between these two meals over the four-hour period (Figure 1B).

### Desire to eat and prospective intake

IO consumption reduced desire to eat significantly more than the RTEC over the four hour period (AUC: IO: 9129.38 ± 884.33 mm × min versus RTEC: 7064.69 ± 886.3 mm × min, p = 0.01), at 60 minutes (p < 0.01), and at 120 minutes (p = 0.01) (Figure 1C). Prospective intake, was significantly lower after both IO and SO consumption compared to the RTEC over the four-hour period (AUC IO: 7968.55 ± 769.24 mm × min, p < 0.01, versus RTEC, SO: 6954.12 ± 769.26 mm × min, p = 0.04 versus RTEC; RTEC: 5525.98 ± 771.02 mm × min). IO consumption reduced prospective intake at 30 minutes (p = 0.01), 60 minutes (p < 0.01) and 120 minutes (p < 0.01) more than consumption of the RTEC. SO consumption also decreased prospective intake at 30 minutes (p = 0.02) and 120 minutes (p = 0.02) more than consumption of the RTEC. IO consumption lowered prospective intake at 60 minutes more than SO (p = 0.02), but prospective intake was not significantly different between the two over the four-hour period (Figure 1D).

### Kinetics of glucose release

The kinetics of starch digestion and glucose release were not significantly different among the three breakfast cereals (IO: 99 ± 3 g × min, SO: 100 ± 3 g × min, RTEC: 98 ± 2 g × min, p > 0.05).

### Physicochemical characteristics of β-glucan

**Figure 2:**
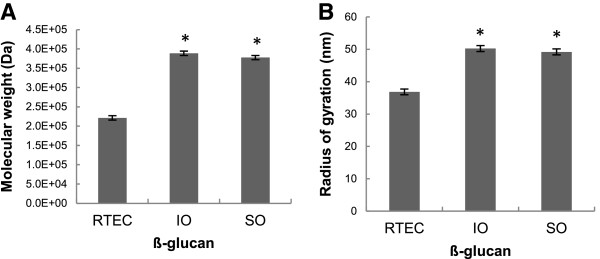
**Least squares means of the molecular weight (Mw) in Daltons (Da) (A) and radius of gyration (Rg) in nanometers (nm) (B), of the β-glucan content of instant oatmeal (IO), old fashioned oatmeal (SO) and the ready-to-eat breakfast cereal (RTEC).** Both varieties of oatmeal had higher molecular weight and radius of gyration that the RTEC (p < 0.01). Values are mean ± standard error.

The molecular weight of β-glucan was higher in both varieties of oatmeal than in the RTEC (IO: 3.89×10^5^ ± 5.46×10^3^ Da, SO: 3.78×10^5^ ± 5.46×10^3^ Da, RTEC: 2.21×10^5^ ± 5.46×10^3^ Da (p < 0.01[IO versus RTEC], p < 0.01[SO versus RTEC]) (Figure 2A). Additionally, the hydrated β-glucan molecules in oatmeal formed larger spheres than those in the RTEC as the radius of gyration was 50.23 ± 0.90 nm for IO, 48.2 ± 0.90 nm for SO, and 36.83 ± 0.90 nm for the RTEC (p < 0.01 [IO versus RTEC] and p < 0.01 [SO versus RTEC]) (Figure 2B).

### Meal viscosities

**Figure 3:**
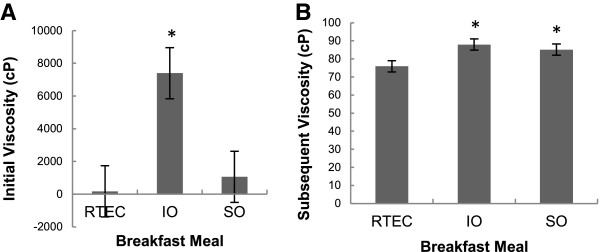
**Mean viscosities of oatmeal and ready-to-eat-breakfast cereal (RTEC) meals observed at the *in vitro *simulation of digestion.** Viscosity values are the means of three replicates and expressed in centipoise (cP) ± standard error. Instant oatmeal (IO) exhibited a higher viscosity than old fashioned oatmeal (SO) (p = 0.03) and the RTEC (p = 0.02) after oral and initial gastric digestion at time = 0 **(A)**. IO (p = 0.01) as well as SO (p < 0.05) demonstrated significantly greater viscosity than the RTEC during the remainder of the in vitro gastric simulation process **(B)**.

IO (7397.17 ± 1564.51 centipoise) exhibited a higher initial viscosity (time = 0) than SO (1063.33 ± 1564.51 centipoise;) and RTEC (175.17 ± 1564.51 centipoise, after oral and initial gastric digestion (p = 0.03 [IO versus SO] and p = 0.02 [IO versus RTEC]) (Figure 3A). IO (87.92 ± 3.12 centipoise) and SO (85.13 ± 3.12 centipoise) demonstrated a greater subsequent viscosity (time ≠ 0) than the RTEC (75.92 ± 3.12 centipoise) during the remainder of the in vitro gastric simulation process (p = 0.01 [IO versus RTEC] and p **<** 0.05 [SO versus RTEC]) (Figure 3B).

## Discussion

Consumption of dietary fiber has been shown to increase satiation and satiety and have a modest effect on long term weight loss [4,5]. In this study, the satiety effects, β-glucan characteristics, and meal viscosities of three different oat-based breakfast cereals were assessed. IO consumption increased fullness, suppressed desire to eat, and reduced prospective intake more than the RTEC did over a four-hour period, and consistently at the 60 minute time-point. SO consumption reduced prospective intake more than the RTEC did, but did not significantly improve any other satiety measures. The content, molecular weight, and radius of gyration of β-glucan in both oatmeal varieties were higher compared to the RTEC, possibly contributing to greater viscosity of the oatmeal types.

Food processing may influence satiety by changing the viscosity and physicochemical properties of β-glucan. Viscosity (η) is a function of concentration (*c*) and molecular weight (*Mw*) of the polymers (η $\propto cM_{w}^{\alpha }$ where α is a parameter depending of the shape of the polymer) [33]. Mechanical processing or excessive heat treatments can change the β-glucan structure reducing its molecular weight and viscosity. Extrusion, a process often used in the production of breakfast cereals can affect the physicochemical properties of the fiber based on the processing techniques employed, and the composition of the ingredients [34]. Both sugar and salt increase the apparent viscosity of β-glucan solutions [35]. Although the oatmeal had no sodium and no added sugar it had a higher molecular weight, radius of gyration, and viscosity than the RTEC, further indicating that the functional properties of β-glucan vary among products.

A serving size (150 kcals) of IO which was composed of thinly cut flakes increased all indicators of satiety, except hunger, compared to an isocaloric oat-based RTEC. However SO, which consisted of thicker flakes only decreased prospective intake compared to the RTEC. Using echo-planar magnetic resonance imaging, Hoad et al. [36]. showed that satiety increases as initial viscosity of the meal increases. Initial viscosity likely modulates a cephalic phase effect in which the orosensory factors play an important role in the overall satiety response. Previous research shows that intestinal infusion of a soup produces a weak effect on the control of appetite, which is progressively amplified with gastric and oral stimulation [37]. In the present study, IO had greater initial and subsequent viscosities compared to the RTEC, whereas SO only had greater subsequent viscosity compared to the RTEC. Thus, the greater initial viscosity of the IO may have increased oral stimulation and produced a greater satiety effect than SO, suggesting that regulation of appetite works in concert with oral, gastric, intestinal, and post-absorptive mechanisms.

It is likely that the thinly cut IO flakes hydrated more easily with the addition of boiling water compared to the thicker SO flakes and may explain why the two oatmeal varieties displayed different viscosities when they first entered the stomach as estimated in the *in vitro* simulation. The addition of oat bran (4 or 8 g β-glucan) to biscuits and juice (enriched biscuits and enriched juice) increased satiety compared to the control meal without β-glucan, but, β-glucan added only to biscuits (enriched biscuits and juice) did not produce this effect [38]. In a comparison between two cereals, oatmeal (2.6 g β-glucan) prepared with hot water produced greater viscosity, larger hydration molecules, and increased satiety compared to a ready-to-eat oat based cereal (1.7 g β-glucan) served with cold milk [19]. Thus, sufficient hydration of the fiber is important for inducing the process of satiety.

Our previous study shows that larger portion sizes of IO and SO (250 kcals, 2.6 - 2.7 g β-glucan) increase satiety more than isocaloric servings of the RTEC (1.7 g β-glucan), particularly in the two- to four- hour period following consumption (CJ Rebello, et al.; manuscript under review). Although we also found that IO and SO consumption increased some satiety measures in the current study, the effects were not as robust perhaps due to the smaller portion sizes. The low volume of food may have caused minimal stomach distension and quickly emptied from the stomach, and the energy content was perhaps insufficient for showing significant differences in satiety at all of the time-points past 60 minutes. Thus, portion size likely plays an important role in detecting satiety differences between two foods within a given time frame.

The effects of β-glucan on appetite and satiety have been assessed in several studies but the results have been inconsistent [14-18,39-42]. In a study investigating the effects of β-glucan on satiety it was shown that consumption of 4 g oat β-glucan served with yogurt, had no effect on satiety despite a reduction in the post prandial blood glucose response [17]. Beck *et al.* concluded that the optimal dose of β-glucan affecting satiety and other markers of appetite regulation were between 4 and 6 g and that the hormonal effects (peptide YY) were mediated through increased viscosity observed with increasing the concentration of β-glucan [18]. However, varying doses from 2.16 g to 5.68 g of oat β-glucan also increased satiety in a dose dependent manner [40]. Thus, the differences in the β-glucan content of a food, in addition to structural and functional differences of the fiber in different food products may influence satiety responses.

The sugar content of the oatmeal breakfast meals was lower than the RTEC breakfast meal. A sensory evaluation of the two breakfast meals was not conducted in this study to determine the palatability of the two test meals. Palatability is not a fixed property of a food. Rather, it is a momentary evaluation liable to change with the experience [43]. Moreover, it appears to affect satiation (meal termination) more than satiety (prolongation of the interval between meals) [44]. While the sweetness of sugar is strongly hedonically positive and may stimulate eating rate, sugars in the gut could generate negative as well as positive feedback signals to influence satiation and satiety [45,46]. Adults do not always equate good taste with sweetness, and their taste preferences are not always direct predictors of appetite regulation [47].

Differences in viscosity arising from differences in physicochemical properties may influence the glycemic response [48]. However, the results obtained from the study of the kinetics of starch digestion and glucose release of the breakfast meals were not significantly different among the three cereals. *In vitro* studies of starch kinetics do not fully reflect the effects of viscosity, stomach motility, or nutrient interactions; but, they permit standardization of conditions. Although oatmeal has been shown to be a food with a high glycemic index [49] there may have been differences in the glycemic indices of oatmeal and the RTEC. However, studies that investigated the effect of the glycemic response on satiety have shown inconsistent results [50-53].

Oatmeal had higher protein content than the RTEC and protein-induced satiety has been demonstrated in several studies. In a study comparing a high protein meal (25% of energy) with a low protein meal (10% of energy) it was found that satiety significantly increased after the high protein meal [54]. In a comparison between breakfast skippers and those who ate a high protein breakfast (35 g protein, 40% of energy content) or a normal protein breakfast (13 g protein, 15% of energy content), both the protein breakfasts increased satiety compared to the breakfast skippers with the high protein breakfast meals eliciting a greater satiety response than did the normal protein breakfast [55]. These studies [54,55], compared meals or diets that differed by 15% to 25% in their energy content from protein. In the present study, the difference in protein content was 2.3 g or 4% of total energy, which is less than the proportion previously shown to increase satiety. Thus, the content and functionality of β-glucan likely influenced the satiety differences observed between the oatmeal types and the RTEC more than the differences in the protein content.

Hormones, neuropeptides, and the glycemic response following consumption of the breakfast meals were not measured in this study. Post-prandial measurements of glucose and endocrine markers of satiety may have helped to clarify the physiologic mechanisms influencing appetite responses, and provided additional support to the conclusions. In a previous study we showed that energy intake at lunch decreases after eating a larger portion size (250 kcal) of oatmeal at breakfast compared to an isocaloric serving of the RTEC; (Rebello CJ *et al.*, manuscript under review) however, in this study food intake was not measured. Appetite scores measured through VAS can be reproduced and are therefore feasible tools to measure appetite and satiety sensations [23]. Nevertheless, proof of concept would require that effects on energy intake and body weight be assessed in future studies. Further, adults in different subgroups may or may not demonstrate disparate treatment response. Thus, it is of interest to compare treatments in subgroups but there must be a sufficient number of participants within the subgroups to support making valid conclusions from such analyses. Because of the efficiency gained in crossover designs, relatively small sample sizes are usually justified. While this is an advantage for investigating the primary outcome in a diverse sample, the typically small sample employed in this study does not provide adequate power to enable drawing reliable conclusions from subgroup analyses.

## Conclusions

The effects of instant oatmeal on satiety demonstrated in this study are similar to the effects that were observed in a previous study comparing the satiety effects of a larger portion size of instant oatmeal with the oat-based RTEC, indicating that IO suppresses appetite, and increases satiety, over a range of portion sizes. SO consumption was less effective in appetite control than IO was when each was compared with the RTEC. Differences in β-glucan content, hydration, and physicochemical properties among the cereals are likely important factors influencing meal viscosity and therefore satiety. A high initial meal viscosity may be associated with increased satiety. Oatmeal provides a readily available source of viscous soluble fiber, and its consumption may be a means of reducing the motivation to eat at future meals. Replacing less-filling breakfast cereals with oatmeal can be an effective tool for promoting satiety.

## Abbreviations

RTEC: Ready-to-eat breakfast cereal; IO: Instant oatmeal; SO: Old fashioned oatmeal; VAS: Visual analog scales; BMI: Body mass index; Gtot: Total glucose content; AACC: American Association of Cereal Chemists; AUC: Area under the curve; Gt=0: Glucose concentration at the beginning of the intestinal phase.

## Competing interests

Marianne O’Shea, Nicholas Bordenave, Yuhui Shi, and YiFang Chu are employees of PepsiCo R&D Nutrition. Candida Rebello, Corby Martin, William Johnson, Hongmei Han, and Frank Greenway have no conflict of interest.

## Authors’ contributions

WDJ, CKM, YC, MO, NB, and FLG designed research; CJR, CKM, FLG, and NB conducted research; WDJ, HH, and CJR analyzed the data; CJR and NB wrote the paper; CJR, WDJ, CKM, NB, YS, YC, and FLG reviewed and edited the manuscript; CJR had primary responsibility for final content. All authors read and approved the final manuscript.
